# Disability and intimate partner violence in England: gender stratified analyses of a probability sample survey

**DOI:** 10.1186/s12889-026-28489-5

**Published:** 2026-07-09

**Authors:** Ladan Hashemi, Hannah Manzur, Annie Bunce, Sally McManus

**Affiliations:** 1 Violence and Society Centre, City St George’s, University of London, Rhind Building, St John St, London, EC1R 0JD UK; 2 School of Health and Medical Sciences, City St George’s, University of London, London, UK; 3 National Centre for Social Research, 35 Northampton Square, London, EC1V 0AX UK

**Keywords:** Disability, Impairment, Activities of daily living, Intimate partner violence, Gender

## Abstract

**Background:**

Few prior random probability sample survey spans experience of all forms of intimate partner violence (IPV) among people with different types of impairment in England. This study aimed to compare the 12-month prevalence of four types of IPV (physical, sexual, psychological, and economic abuse) for people with and without four types of impairment (physical, sensory, learning impairments, and experiencing difficulties with activities of daily living (ADLs)), taking account of variations by gender.

**Methods:**

This study used data from the 2014 Adult Psychiatric Morbidity Survey, a cross-sectional survey that covered the household population of England aged 16 years and older. Data was analysed on 7546 respondents (4488 women and 3058 men). Logistic regression was conducted and adjusted odds ratios (AORs) were reported separately for men and women.

**Results:**

Disabled women reported significantly higher rates of psychological (AOR = 1.98, 95% CI = 1.31–2.98) and sexual IPV (AOR = 2.46, 95% CI = 1.55–3.91) than non-disabled women. No significant association was found for physical IPV. Women with a learning impairment reported the highest prevalence for almost all types of IPV. Women reporting restricted ADLs were at higher risk of experiencing economic abuse (AOR = 2.88, 95% CI = 1.33–6.23). Disabled men did not have a higher risk of experiencing any forms of IPV compared with non-disabled men.

**Conclusion:**

While violence may contribute to the onset of some forms of disability, our findings suggest that disability may also enhance the risk of IPV for disabled women, particularly for women with a learning impairment. Investment in targeted violence prevention programs specifically for disabled women is warranted.

**Supplementary Information:**

The online version contains supplementary material available at https://doi.org/10.1186/s12889-026-28489-5.

## Introduction

Intimate partner violence (IPV) against disabled people is a significant public health and human rights issue, yet there has been no random-probability sample study examining associations between different disability types and forms of IPV in England.

Approximately 16% of the world’s population experiences at least one form of chronic impairment [1]. In England, population-based surveys have estimated the prevalence of disability to range between 16.2% and 32.9% [2], varying with the definition of disability used.

The term ‘disability’ is subject to diverse interpretations, with definitions varying according to self-identification, legal frameworks, conceptual models, administrative purposes, or funding criteria. The World Health Organization (WHO) characterises disability as encompassing impairments, activity limitations, and participation restrictions, representing the negative aspects of interaction between individuals with a health condition and personal and environmental factors [3]. Under the UK 2010 Equality Act, ‘disability’ refers to a physical or mental impairment that substantially and long-term adversely affects a person’s ability to perform normal daily activities, where ‘substantial’ indicates effects beyond minor or trivial, and ‘long-term’ indicates effects lasting more than 12 months or that are fluctuating, recurring, or progressive [4].

There is growing recognition that disability heightens vulnerability to gender-based violence (GBV), while violence can lead to or exacerbate severity of disability [5]. IPV is one of the most common forms of GBV, defined as physical, sexual, emotional or psychological, economic violence/abuse or controlling behaviours occurring between former or current intimate partners [6]. The WHO estimates that, globally, 27% of women aged 15–49 have experienced physical and/or sexual IPV in their lifetime [7]. In the UK, the estimated lifetime prevalence of IPV among ever-partnered women aged 15–59 is 24% [7]. 

Worldwide, gender disparities are notable in experiences of IPV. While men also experience IPV, women encounter more frequent physical violence, more severe violence, higher rates of sexual IPV, increased coercive control, greater instances of injury, and heightened fear [8–12]. For example, in England and Wales, Walby and Towers (2017) found that three-quarters (76%) of victims experiencing repeat domestic violent crime were female, and 83% of high-frequency victims of domestic violent crime (10 or more crimes in a year) were female [13].

The dynamics of IPV are diverse and complex for disabled people, as conditions and impairments may be exploited by abusers, and issues of increased power and control over disabled people may multiply their vulnerability and isolation [14, 15]. Impairment may also exacerbate the risk of IPV by diminishing disabled people’s ability to defend themselves, recognise, or report abuse [14, 16]. Limited access to support services [17] and societal attitudes that devalue or stigmatise disabled people may also heighten the risk of IPV [18].

Globally, disabled women experience higher rates of IPV compared to non-disabled women [19, 20]. In the UK, 11.1% of women with a long-term illness or disability experienced past-year partner abuse, compared with 4.9% of non-disabled women [21]. However, the need for improved data has been underscored in systematic reviews as disabled women are often underrepresented in research [19, 20]. This lack of evidence undermines the effectiveness of programs to address their specific needs.

Existing studies have largely focused on experience of “any IPV”, or limited subtypes (predominantly physical and/or sexual IPV), often neglecting psychological and economic abuse [19, 20].

This is important as a recent review reveals variability in findings with only 50% of studies identified a heightened risk of “any IPV” among disabled women compared to non-disabled women, while individual subtypes including financial (100% of studies), physical (81.3%), psychological (78.5%) and sexual (73.3%) consistently showed heightened risk [19]. This highlights the importance of examining different forms of IPV to better understand the relationship between disability and IPV victimisation.

In 2015, a scoping review of evidence identified further research gaps in the UK and globally [16], which remain a decade later. For example, the need for nationally representative studies remains critical as most research has used small samples from service providers’ datasets which lack generalisability and often fail to account for the broader adversities faced by those impacted by disability and/or IPV. Additionally, much of the literature has focused on women only, precluding gender comparisons which would improve understanding of gendered dynamics in disability-related IPV, as well as neglecting men’s experiences of IPV [22]. Whilst the literature on IPV against disabled women emphasises the role of coercive control and intimate partner-carer relations (see, for example [14, 23], , the role of disability for men’s particular experiences of IPV, such as barriers to disclosure and support [24, 25], is largely missing from the literature [22].

The temporal relationship between impairment and IPV also remains poorly understood, partly due to studies focusing on lifetime exposure to IPV (e.g [26]), which leaves the direction of the association unclear and the timeline undefined [16].

Although the population prevalence of disability in England is substantial, there remain no random-probability sample studies that examine the intersection between different disability types and IPV subtypes for both women and men. The only population-based survey that explored this did not specifically report on IPV but focused on the broader concept of domestic (partner or family) versus non-domestic violence among disabled individuals. Moreover, no gender-stratified analyses were reported [27].

The 2014 Adult Psychiatric Morbidity Survey (APMS), a large adult population survey in England, provided a unique opportunity to estimate the prevalence of four types of IPV for women and men with different disability types. This analysis leverages APMS data to enhance our understanding of the disability-IPV nexus, with the following objectives:

1. describe past-year prevalence of physical, sexual, psychological, economic and any IPV by disability status (type and number of limiting impairments).
2. examine associations between disability types and numbers and different forms of IPV, controlling for sociodemographic characteristics.
3. explore gender patterns in IPV by disability status.

## Methods

### Study design and participants

We analysed the 2014 APMS, a cross-sectional probability-sample survey. The APMS is designed to provide England’s National Statistics for monitoring of mental health and treatment access in the general population. The data series is unique and valuable because of the wide range of mental and physical health conditions, substance disorders and self-harm behaviours covered. The survey employs rigorous screening and assessment tools, so that undiagnosed conditions can be identified, including in those not in contact with health services. The main survey is designed to examine temporal change, with waves conducted in 1993, 2000, 2007 and 2014 using consistent methods [28]. Verbal informed consent was obtained from all participants. The current study is a secondary analysis of the 2014 APMS data, focusing on information collected on risk factors for poor mental health, specifically experiences of intimate partner violence and abuse.

The survey covered the household population of England aged 16+, using a stratified, multistage random sampling design. This involved the selection of primary sampling units, addresses within selected primary sampling units, and one English-speaking adult from each address. Fieldwork occurred from May 2014 to September 2015, with verbal informed consent. The achieved sample comprised 7546 individuals, reflecting a 57% response rate.

Interviews were conducted in participant’s homes by trained interviewers, averaging 1.5 h. They involved computer-assisted personal interviewing, with sensitive information collected through computer-assisted self-completion interviews via the interviewer’s laptop. Secondary analyses were approved by (removed to enable blind review). The authors assert that all procedures contributing to this work comply with the ethical standards of the relevant national and institutional committees on human experimentation and with the Helsinki Declaration of 1975, as revised in 2008. Methodological details are published elsewhere [28].

### Sample and analytic sample

The APMS sample included 7546 individuals. Participants with missing data (primarily due to non-completion of the self-completion section, which included questions on IPV experience) were excluded from the regression analysis. Previous analyses of APMS have shown that those who did not complete the self-completion section were significantly older (*p* < 0·0001) than those who did [29]. Non-response to the self-completion section was not associated with gender. Participants who did not respond to IPV items despite completing the self-completion section, or who responded ‘don’t know,’ were also excluded from the regression analysis. The whole APMS sample was used to estimate the prevalence of disability subtypes, as the aim of the Table 1 was to profile these subtypes in the general population and provide a more accurate estimate of the prevalence of different types of disability in England.

**Table 1:** Weighted descriptive analyses of demographic characteristics by disability, Adult Psychiatric Morbidity Survey 2014

		Disability							
		None	Any (One or more disability Types)	Restricted ADLs	Physical disability	Sensory disability	Learning disability	One versus multiple (*n* = 2882)	
								One disability type	Multiple disability types
Characteristics		n(W%)	n(W%)	n(W%)	n(W%)	n(W%)	n(W%)	n(W%)	n(W%)
Total		4664 (65.8)	2882(34.2)	1993 (22.3)	1341 (15.3)	1099 (12.6)	225(3.4)	1549(57.2)	1333(42.8)
Gender	Men	1941 (67.8)	1117(32.2)	756(20.4)	460(12.8)	489(13.2)	123(4.3)	592(57.5)	525 (42.5)
	Women	2723 (63.9)	1765(36.1)	1237(24.1)	881(17.8)	610(12.0)	102(2.5)	957(56.9)	808 (43.0)
P-value^b^		**0.01**		**0.006**	**< 0.001**	**0.004**	**< 0.001**	0.522	
Age group	16–34	1202 (76)	393(24.0)	208 (12.6)	157 (0.09)	95 (6.1)	77 (5.0)	279 (72.9)	114 (27.0)
	35–54	1733 (71.2)	741(28.8)	410 (15.6)	405 (15.3)	235 (9.2)	74 (3.2)	458 (63.5)	283 (36.5)
	55–70	1403 (59.4)	1012(40.6)	730 (28.6)	488 (19.1)	397 (16.5)	62 (2.5)	514 (50.7)	498 (49.3)
	75+	326 (32.2)	736(67.7)	645 (58.8)	291 (26.3)	372 (34.3)	12 (1.1)	298 (40.7)	438 (59.3)
P-value^b^		**< 0.001**		**< 0.001**	**< 0.001**	**< 0.001**	**< 0.001**	**< 0.001**	
Ethnicity	White (British and other white)	4176 (65.2)	2637(34.8)	1813 (22.4)	1227(15.5)	1017 (12.9)	202 (3.5)	1418 (57.3)	1219 (42.7)
	Black/Black British	129 (68.8)	68 (31.2)	53 (24.4)	30 (13.1)	18 (10)	6 (2.2)	39 (56.2)	29 (43.8)
	Asian/Asian British	245 (71.1)	112 (28.9)	80 (19.5)	51 (14.6)	39 (10.2)	8 (1.7)	61(54.8)	51 (45.2)
	Mixed, Multiple, Other	99 (67.7)	52 (32.4)	37 (23.0)	23 (11.7)	18 (10.6)	7 (3.3)	28 (62.9)	24 (37.0)
P-value^b^		**0.020**		0.336	0.190	**0.018**	0.548	0.949	
Housing tenure	Owner occupied	3190 (68.1)	1731 (31.9)	1140 (19.9)	757 (13.6)	685 (12.3)	77 (0.02)	1004 (60.6)	727 (39.4)
	Private or other	904 (73.5)	400 (26.5)	248 (14.9)	188 (12.0)	130 (8.5)	41 (3.1)	238 (63.8)	162 (36.2)
	Social renter	541 (46.9)	729 (53.0)	590 (41.2)	384 (26.2)	273 (18.7)	100 (9.1)	299 (45.2)	430(54.8)
p-value^b^		**< 0.001**		**< 0.001**	**< 0.001**	**< 0.001**	**< 0.001**	**< 0.001**	
Neighbourhood deprivation ^c^	Least deprived areas	2102 (71.8)	1002 (28.2)	646 (17.0)	443 (11.9)	406 (11.2)	56 (2.0)	574 (60.6)	428 (39.3)
	Moderately deprived area	942 (64.7)	621 (35.3)	434 (23.4)	287 (15.9)	237 (12.7)	36 (2.3)	339 (58.9)	282 (41.1)
	Most deprived areas	1620 (60.4)	1259 (39.6)	913 (27.0)	611 (18.4)	456(13.9)	133 (5.3)	636 (54.0)	623 (46.0)
p-value^b^		**< 0.001**		**< 0.001**	**< 0.001**	**0.008**	**< 0.001**	**0.005**	
Serious debt in past year	Yes	264 (46.2)	327 (53.8)	237 (37.3)	165 (25.7)	102 (16.7)	60 (10.3)	160 (53.3)	167 (46.7)
	No	4375 (67.5)	2526 (32.5)	1737 (21.0)	1161 (14.5)	981 (12.1)	153 (2.6)	1378 (57.8)	1148 (42.2)
p-value^b^		**< 0.001**		**< 0.001**	**< 0.001**	**0.042**	**< 0.001**	< 0.055	
Marital status	Married/cohabitating	2812 (68.6)	1325 (31.4)	802 (18.6)	629 (14.6)	486 (11.4)	98 (2.9)	779 (59.4)	546 (40.6)
	Single	1039 (70.0)	549 (29.9)	370 (19.2)	234 (11.2)	176 (9.2)	83 (5.0)	319 (64.8)	230 (35.2)
	Divorced/separated/widow	813 (45.8)	1008 (54.2)	821 (43.8)	478 (25.6)	437 (23.9)	44 (2.5)	451 (44.3)	557 (55.7)
p-value^b^		**< 0.001**		**< 0.001**	**< 0.001**	**< 0.001**	**< 0.001**	**< 0.001**	

^a^ Adults aged 16 and over living in households in England, Adult Psychiatric Morbidity Survey 2014

^b^ p-value from chi square test examining the association between each characteristic and reported disability in the past 12 months preceding data collection. Boldface indicates significant association (*p* < 0.05)

^c^ Least deprived areas comprise the two least deprived quintiles and most deprived areas comprise the two most deprived quintiles, based on ranking of area-level English Index of Multiple Deprivation scores

W%: Percentages weighted to reflect survey design. Percentages shown are column percentages. The “Multiple disability types” category may include respondents who are counted in more than one disability group (physical, sensory, learning, or ADL), reflecting overlap between these measures

### Measures

#### Disability

A broad definition was used, including four self-reported aspects: need assistance with activities of daily living (ADL), limiting long-term physical health conditions, sensory (sight or hearing) impairment, and learning impairment. This approach aligns with the World Health Organization’s definition of disability as encompassing impairments, activity limitations, and participation restrictions, which recognises the interaction between health conditions and environmental factors [30].

We created a binary variable for experiencing one or more impairment types (“any disability”) versus none, and a separate variable distinguishing participants with one impairment versus two or more (“multiple disabilities”). Specific disability categories (Restricted ADLs, physical, sensory, learning) are nested within the “any disability” group, meaning all participants counted in the individual categories are also included in “any disability.” Here, the term “disability types” refers to disability associated with specific impairments or health conditions. For survey questions that included disability measures see Appendix eTable 1.

In this paper, we adopt concerns articulated by the social model of disability and use terminology informed by it [31] while aligning with a biopsychosocial model approach. In the UK, the term ‘disabled people’ is generally preferred, reflecting the understanding that individuals with impairments are ‘disabled’ by societal barriers [31]. This approach is widely supported by disability advocacy groups in the UK, including *Scope* [32].

#### Demographic and socioeconomic characteristics

Participants self-reported gender (women, men), age (banded for analysis), and marital or cohabitation status (single; married or cohabiting; or separated, divorced, or widowed). Ethnicity was self-ascribed and grouped into White British; White other; Black or Black British; Asian or Asian British; and mixed, multiple, or other.

Socioeconomic context included housing tenure (owner-occupier, renting from a social landlord, or renting from a private landlord) and being in serious debt (yes, no). Area-level deprivation was measured using quintiled English Index of Multiple Deprivation scores [33]. The selection of covariates for inclusion in the multivariable models was informed by literature indicating associations with both disability and IPV.

#### IPV

Experience of physical, sexual, economic, and psychological IPV from a current or former partner was asked about in the self-completion interview section. These questions were adapted from the Crime Survey for England and Wales (CSEW), which originally developed its items based on the Conflict Tactics Scale (CTS) [34]. However, the CSEW uses a modified version of the scale tailored for large population surveys, distinct from the revised CTS2. Variables were created for 12-month experience of each IPV type and of any IPV. An intimate partner was defined as any current or previous boyfriend, girlfriend, husband, wife, or civil partner. Participants with no previous partners were coded as having not experienced IPV. Appendix eTable 1 provides the IPV questions used. Missing data including: ‘do not know’ and ‘refused’ were excluded from all analyses. Less than 2% of any variable had missing data.

#### Statistical procedure

Analyses were conducted in Stata/SE 17.0 [35]. Survey weights, clustering, and stratification were accounted for using the Stata svy command to correctly adjust for the complex survey design and non-response. The full weighting strategy is detailed in the survey reports [36].

Weighted proportions described the prevalence of disability subtypes by sociodemographic characteristics (Table 1) and by exposure to each, any, and multiple IPV types (Table 3) stratified by gender. Chi-square tests were used to determine whether there were differences in the distribution of each disability subtype, any disability, and multiple disability types across sub-groups separately for men and women (Table 1).

Multivariable logistic regressions calculated the odds ratios (OR) for experiencing any IPV and different IPV types for those reported any, different disability types, and multiple disability types experienced for the whole sample (Table 2) and separately for men and women, adjusting for age, ethnicity and socioeconomic factors (Table 5). Unadjusted OR were also calculated and reported (Table 4). Sensitivity analyses determined that housing tenure, neighbourhood deprivation, and serious debt sufficiently captured socioeconomic context. Results are reported with 95% confidence intervals and statistical significance set at p-value < 0.05.

**Table 2:** Weighted descriptive analysis and associations of 12-month IPV by disability status, type, and number in the whole sample, Adult Psychiatric Morbidity Survey 2014

	Physical IPV		Sexual IPV		Psychological IPV		Economic IPV		Any IPV	
	*n*(W%)	AOR (95% CI)	*n*(W%)	AOR (95% CI)	*n*(W%)	AOR (95% CI)	*n*(W%)	AOR (95% CI)	*n*(W%)	AOR (95% CI)
No disability (ref)	65 (1.5)	Ref	71 (1.2)	Ref	122(2.6)	Ref	31 (0.6)	Ref	221 (4.6)	Ref
At least one disability	38 (1.5)	1.06 (0.65–1.73)	83 (2.8)	**2.19 (1.41–3.40)**	111(4.4)	**1.55 (1.13–2.12)**	33 (1.2)	**1.87 (1.04–3.35)**	193 (7.5)	**1.61 (1.27–2.05)**
Restricted ADLs	29 (1.7)	1.43 (0.85–2.41)	53 (2.5)	1.52 (0.97–2.38)	72 (4.3)	**1.60 (1.14–2.24)**	26 (1.6)	**2.60 (1.37–4.93)**	125 (7.3)	**1.62 (1.24–2.1)**
Physical disability	16 (1.0)	0.69 (0.37–1.31)	50 (3.7)	**2.38 (1.47–3.85)**	56 (4.5)	**1.59 (1.08–2.33)**	17 (1.1)	1.30 (0.71–2.38)	102 (8.0)	**1.62 (1.22–2.15)**
Sensory disability	13 (1.5)	1.28 (0.53–3.12)	32 (2.5)	**1.63 (1.02–2.60)**	39 (3.7)	1.44 (0.97–2.13)	5 (0.3)	0.29 (0.10–0.87)	70 (6.5)	**1.46 (1.05–2.03)**
Learning disability	5 (2.3)	0.75 (0.19–3.01)	10 (3.9)	2.33 (0.84–6.46)	21 (11.2)	**2.16 (1.10–4.23)**	4 (2.6)	2.47 (0.67–9.16)	27 (13.7)	1.76 (0.98–3.16)
One disability type	18 (1.5)	Ref	40 (2.5)	Ref	57 (3.9)	Ref	17 (1.2)	Ref	99 (7.0)	Ref
Multiple disability types	20 (1.6)	1.37 (0.54–3.52)	43 (3.2)	1.50 (0.83–2.70)	54 (5.0)	**1.67 (0.99–2.82)**	16 (1.3)	1.14 (0.47–2.76)	94 (8.1)	**1.48 (1.00-2.19)**

AORs: Odds ratios adjusted for gender, age, ethnicity, marital status, housing tenure, area-level deprivation, and any serious debt. Boldface indicates significant association (*p* < 0.05)

W%: Percentages weighted to reflect survey design. Percentages shown are column percentages

The “Multiple disability types” category may include respondents who are counted in more than one disability group (physical, sensory, learning, or ADL), reflecting overlap between these measures

## Results

The APMS sample included 7546 individuals. A total of 462 participants were excluded from the regression analysis due to missing data, primarily non-completion of the self-completion section. A further 20 participants did not respond to IPV items despite completing the self-completion section, and 6 responded ‘don’t know’; these were also excluded. This yielded an analytic sample of 7058 for the regression analyses, while the full sample (*n* = 7546) was retained for the disability prevalence estimates reported in Table 1.

Table 1 describes the prevalence of disability subtypes (any disability, ADLs, physical disability, sensory disability, learning impairment, and count of disability types, one versus multiple) for the whole sample and stratified by sociodemographic characteristics. The prevalence of any disability among the whole sample was 34.2%. The most common type of disability was restricted ADLs reported by 22.4% of the sample, followed by physical health disability (15.3%). The least common type of disability was learning impairment (3.4%). Disability was more prevalent in women (36.1%) than in men (32.2%, *p* < 0.01), more prevalent among those living in the most deprived areas (39.6%) than those in the least deprived areas (28.2%, *p* < 0.001), and more prevalent in people who were divorced, separated or widowed (54.2%) than married people (31.4%, *p* < 0.001).

Disability was also more prevalent in social renters (53%) than in homeowners (31.90%, *p* < 0.001), and in those with serious debt (53.8%) than those without (32.5%, *p* < 0.001). Older age groups were over-represented among those with disability compared with younger age groups for all types of disability (*p* < 0.001). The only exception was learning impairment which was more prevalent among younger people (5.0% in those aged 16 − 34 years versus 1.1% in those aged 75 + years, *p* < 0.001). Regarding ethnicity and disability, at least one type (any disability) and sensory disability were more prevalent among those identified as White (British and other White) than among those from other ethnic groups. Of those with multiple disability types, a larger proportion were aged 75+, renting from a social landlord, living in most deprived areas, and divorced, separated or widowed (*p* < 0.001) (Table 1).

Regarding the prevalence of 12-month IPV, irrespective of disability status, psychological IPV was the most common form of IPV reported by 3.5% of women and 2.3% of men. Women had higher odds of experiencing sexual (OR: 20.65, 95%CI 8.2–52.1), psychological (OR: 1.53, 95% CI 1.12–2.08), and any IPV (OR = 2.01, 95%CI 1.58–2.56) in the past year than men (not shown in the tables).

### 12-month prevalence of IPV by disability status for the whole sample

Table 2 describes prevalence rate of each IPV types and any IPV by disability status (with versus without any disability), disability type, and disability count (one disability type versus multiple disability types) for the whole sample. The findings are presented as the number of participants in each group and weighted percentage (n & W%). The results of logistic regression modelling examining association between disability and IPV are also presented.

Disabled people, regardless of gender, had higher odds of reporting sexual, psychological, economic, and any form of IPV compared to non-disabled people. (Table 2). Those with multiple types of disability had higher odds of reporting psychological IPV and any IPV compared to those with a single disability type (Table 2).

### 12-month prevalence of IPV by disability status separately for women and men

Table 3 describes the prevalence rates of IPV among individuals with disability, stratified by gender. When comparing genders, disabled women were more likely to report IPV than non-disabled women. Disabled men were not more likely to report IPV than non-disabled men (Table 3). Almost 10% of disabled women reported experience of at least one form of IPV in the past 12 months. This was 5.5% for non-disabled women and 3.2% for non-disabled men (Table 3).

**Table 3:** Weighted descriptive analysis of different types of past 12-month IPV and any IPV by disability status, type, and number, stratified by gender, Adult Psychiatric Morbidity survey 2014, England

	IPV									
	Physical IPV		Sexual IPV		Psychological IPV		Economic IPV		Any IPV	
	*n* (W%)		*n* (W%)		*n* (W%)		*n* (W%)		*n* (W%)	
	**Women**	**Men**	**Women**	**Men**	**Women**	**Men**	**Women**	**Men**	**Women**	**Men**
Total	66 (1.5)	37 (1.2)	148 (2.9)	6 (0.1)	165 (3.5)	68 (2.3)	47 (0.9)	17 (0.6)	316 (6.7)	98 (3.4)
No disability	37 (1.4)	28 (1.6)	67 (2.1)	4 (0.16)	81 (2.9)	41 (2.1)	24 (0.8)	7 (0.4)	158 (5.5)	63 (3.25)
At least one disability type	29 (1.7)	9 (1.3)	81 (5.0)	2 (0.1)	84 (5.3)	27 (3.3)	23 (1.3)	10 (1.1)	158(10.0)	35 (4.4)
Restricted ADLs	23 (2.0)	6 (1.4)	51 (4.4)	2 (0.2)	52 (5.0)	20 (3.3)	19 (1.7)	7 (1.3)	101 (9.6)	24 (4.4)
Physical disability	14 (1.4)	2 (0.5)	49 (6.1)	1 (0.1)	43 (5.2)	13 (3.5)	13 (1.1)	4 (1.0)	86 (10.6)	16 (4.3)
Sensory disability	9 (1.1)	4 (1.9)	30 (4.8)	2 (0.3)	29 (5.1)	10 (2.4)	3 (0.3)	2 (0.4)	57 (9.7)	13 (3.3)
Learning disability	2 (1.2)	3 (2.9)	9 (9.2)	1 (0.6)	12 (14.6)	9 (9.1)	1 (1.9)	3 (3.0)	18 (20.9)	9 (9.3)
One disability type	12 (1.5)	6 (1.5)	40 (4.6)	0 (0)	44 (4.7)	13 (2.7)	11 (1.2)	6 (1.1)	79 (8.9)	20 (4.8)
Multiple disability types	17 (2.1)	3 (0.9)	41 (5.6)	2 (0.3)	40 (6.1)	14 (3.1)	12 (1.4)	4 (1.2)	79 (11.6)	15 (3.9)

W%: Percentages weighted to reflect survey design. Percentages shown are column percentages

The “Multiple disability types” category may include respondents who are counted in more than one disability group (physical, sensory, learning, or ADL), reflecting overlap between these measures

Psychological IPV was the most prevalent form of 12-month IPV, reported by 5.3% of disabled women and 3.3% of disabled men. This was followed by sexual IPV, (5.0% of disabled women, 0.1% of disabled men) (Table 3). When considering the type of impairment, women with a learning impairment had the highest prevalence for almost all types of IPVs; 20.9% of women with a learning impairment experienced past-year IPV (any type). This was 9.3% for men with a learning impairment (Table 3).

### Association between disability and IPV

Table 4 presents the results of logistic regression modelling, showing the unadjusted association between disability and IPV, stratified by gender.

Disabled women had higher odds of experiencing all types of IPV during past year as non-disabled women (AORs ranged from 1.79 to 2.93). The exceptions were for physical and economic IPV, where no significant difference was found for women with and without impairments (Table 4). For men, there were no significant differences in the prevalence of any forms of past-year IPV between disabled and non-disabled men, except for economic IPV (Table 4). Men with learning impairment had higher odds of reporting almost all types of IPV and any IPV (except for physical IPV). However, these associations did not remain significant after adjustment for socioeconomic covariates (see Table 5). No differences were found between disabled people with two or more impairments and those with one, for either women or men.

**Table 4:** Logistic regression modelling findings on the association between disability status, type, and number, and types of 12-month IPV, stratified by gender (unadjusted)

	Physical IPV		Sexual IPV		Psychological IPV		Economic IPV		Any IPV	
	OR (95% CI)		OR (95% CI)		OR (95% CI)		OR (95% CI)		OR (95% CI)	
	Women	Men	Women	Men	Women	Men	Women	Men	Women	Men
No disability (ref)	**Ref**	**Ref**	**Ref**	**Ref**	**Ref**	**Ref**	**Ref**	**Ref**	**Ref**	**Ref**
At least one disability	1.20 (0.67–2.18)	0.78 (0.39–1.59)	**2.38 (1.62–3.49)**	0.82 (0.13–5.12)	**1.79 (1.23–2.61)**	1.54 (0.94–2.51)	1.54 (0.82–2.88)	**2.93 (1.03–8.35)**	**1.81 (1.39–2.36)**	1.32 (0.88–1.99)
Restricted ADLs	1.44 (0.75–2.75)	0.90 (0.43–1.87)	**1.58 (1.07–2.34)**	1.54 (0.25–9.58)	**1.47 (1.02–2.12)**	1.47 (0.85–2.55)	**2.22 (1.16–4.22)**	**2.95 (1.01–8.60)**	**1.51 (1.15–1.99)**	1.24 (0.79–1.96)
Physical disability	0.87 (0.44–1.70)	0.28 (0.06–1.30)	**2.48 (1.58–3.89)**	0.94 (0.10–8.88)	1.51 (0.99–2.31)	1.53 (0.80–2.91)	1.17 (0.63–2.18)	1.74 (0.54–5.68)	**1.67 (1.22–2.28)**	1.19 (0.66–2.15)
Sensory disability	1.27 (0.56–2.88)	0.68 (0.22–2.13)	**2.48 (1.58–3.89)**	0.94 (0.10–8.88)	1.43 (0.90–2.29)	0.94 (0.43–2.07)	0.27 (0.08–0.87)	0.61 (0.13–2.82)	**1.44 (1.04–2.01)**	0.87 (0.45–1.71)
Learning disability	0.77 (0.18–3.25)	2.06 (0.46–9.23)	**3.26 (1.26–8.43)**	**4.27 (0.44–41.07)**	**4.68 (2.07–10.58)**	**4.42 (1.75–11.20)**	2.00 (0.27–14.88)	**5.91 (1.39–25.05)**	**3.55 (1.80–6.97)**	**2.82 (1.13–7.06)**
One disability type	**Ref**	**Ref**	**Ref**	**Ref**	**Ref**	**Ref**	**Ref**	**Ref**	**Ref**	**Ref**
Multiple disability types	1.44 (0.51–4.07)	0.62 (0.06–6.37)	1.22 (0.71–2.11)	†	1.32 (0.78–2.22)	1.22 (0.50–2.96)	1.15 (0.48–2.73)	1.05 (0.19–5.92)	1.34 (0.92–1.93)	0.80 (0.31–2.02)

ORs: Odds ratios (unadjusted). Boldface indicates significant association (*p* < 0.05)

†: Odds ratio could not be calculated due to zero observations in the reference or comparison group or sparse data

The “Multiple disability types” category may include respondents who are counted in more than one disability group (physical, sensory, learning, or ADL), reflecting overlap between these measures.

**Table 5:** Logistic regression modelling findings on the association between disability status, type, and number, and types of 12-month IPV, stratified by gender (adjusted)

	Physical IPV		Sexual IPV		Psychological IPV		Economic IPV		Any IPV	
	AOR (95% CI)		AOR (95% CI)		AOR (95% CI)		AOR (95% CI)		AOR (95% CI)	
	Women	Men	Women	Men	Women	Men	Women	Men	Women	Men
No disability (ref)	Ref	Ref	Ref	Ref	Ref	Ref	Ref	Ref	Ref	Ref
At least one disability	1.32 (0.69–2.52)	0.75 (0.33–1.69)	**2.46 (1.55–3.90)**	0.24 (0.09–0.65)	**1.97 (1.31–2.97)**	1.12 (0.65–1.92)	1.77 (0.89–3.54)	2.11 (0.79–5.62)	**1.96 (1.45–2.64**)	1.16 (0.73–1.83)
Restricted ADLs	1.99 (0.92–4.28)	0.88 (0.41–1.87)	**1.70 (1.07–2.67)**	0.50 (0.18–1.38)	**2.00 (1.33–3.02)**	1.34 (0.75–2.40)	**2.95 (1.38–6.30)**	2.17 (0.74–6.33)	**1.88 (1.37–2.59)**	1.33 (0.82–2.17)
Physical disability	0.29 (0.06–1.47)	0.84 (0.40–1.75)	**2.45 (1.48–4.05)**	0.87 (0.31–2.43)	**1.62 (1.01–2.59)**	1.45 (0.65–3.21)	1.27 (0.69–2.36)	1.26 (0.30–5.24)	**1.72 (1.22–2.43)**	1.29 (0.65–2.54)
Sensory disability	1.85 (0.81–4.23)	0.73 (0.16–3.37)	**1.73 (1.05–2.83)**	0.94 (0.31–2.88)	**2.00 (1.20–3.35)**	0.92 (0.41–2.05)	0.30 (0.09–0.97)	0.25 (0.03–2.02)	**1.82 (1.26–2.64)**	1.02 (0.49–2.11)
Learning disability	0.36 (0.08–1.64)	1.13 (0.18–7.05)	2.54 (0.86–7.46)	†	2.36 (0.88–6.33)	1.99 (0.74–5.36)	1.56 (0.18–13.6)	3.04(0.57–16.1)	**2.19 (1.01–4.79)**	1.35 (0.50–3.65)
One disability type	Ref	Ref	Ref	Ref	Ref	Ref	Ref	Ref	Ref	Ref
Multiple disability types	2.12 (0.71–6.31)	0.66 (0.05–8.31)	1.48 (0.80–2.71)	†	**1.86 (1.01–3.45)**	1.29 (0.48–3.48)	1.37 (0.54–3.47)	0.85 (0.12–6.08)	**1.79 (1.17–2.74)**	0.86 (0.33–2.21)

AORs: Odds ratios adjusted for age, ethnicity, marital status, housing tenure, area-level deprivation, and any serious debt. Boldface indicates significant association (*p* < 0.05)

†: Odds ratio could not be calculated due to zero observations in the reference or comparison group or sparse data

The “Multiple disability types” category may include respondents who are counted in more than one disability group (physical, sensory, learning, or ADL), reflecting overlap between these measures

Table 5 presents the results of logistic regression modelling on the association between disability and IPV, stratified by gender and adjusted for covariates.

Controlling for age and socioeconomic characteristics, disabled women had almost twice the odds of experiencing all types of IPV during past year as non-disabled women (AORs ranged from 1.96 to 2.46). The exceptions were for physical and economic IPV, where no significant difference was found for women with and without impairments (Table 5). For men, there were no significant differences in the prevalence of any forms of past-year IPV between disabled and non-disabled men (Table 5).

A similar gender pattern was found for those with two or more impairments versus those with one. For disabled women, having two or more impairments increased the risk of experiencing any form of IPV compared with having one. However, differences were not significant for men with two or more impairments compared with men with one (Table 5).

## Discussion

Using a robust national probability sample of women and men, this study analysed the prevalence of and associations between disability and IPV. The results provide new evidence of the compounding and varying effects of gender and disability on IPV victimisation rates in England. We found a gendered association between experiences of disabilities and IPV, with disabled women having nearly twice the odds of experiencing IPV compared to non-disabled women. In contrast, disability among men was not significantly associated with increased odds of IPV victimisation.

The finding of higher prevalence of IPV in women compared to men in this study is supported by a large body of literature on the gendered nature of IPV [8, 9, 37, 38].

### Association between disability and IPV and gender differences

We found that disabled people had higher odds of experiencing IPV in the past 12 months compared to non-disabled people. This pattern was consistent across all types of disability and non-physical forms of IPV. Consistent with previous studies [26, 39, 40], we found a significant association between disability and risk of IPV victimisation among women, but, inconsistent with these studies, not for men.

These findings align with the ecological framework and intersectionality theory in explaining how disability and gender intersect to shape IPV risk. The ecological framework highlights how individual, relational, and societal factors interact to increase vulnerability [41], while intersectionality shows how interacting social inequalities, like gender and disability, create unique risks [42, 43]. Disabled women face elevated risk of IPV due to factors such as dependency and social invisibility, particularly within gendered care and intimate-partner relationships. In contrast, disabled men may experience IPV differently, shaped by gendered power dynamics, social expectations, and distinct barriers to disclosure and accessing support [24, 25, 44]. Our findings, consistent with these theories, underscore the complex, multi-level influences on IPV victimisation.

These findings provide new evidence of the association between gender, disability and IPV in the UK context, including gendered associations between specific IPV and disability types. Further, this study contributes towards a deeper understanding of the role of social and contextual factors in how disability impacts people’s lived experiences and social relations. The social model of disability holds social and environmental contexts as critical for how impairments impact individuals’ experiences, where disability arises from non-supportive and unequal environments rather than individuals’ impairments. Social and contextual factors are important for understanding disabled people’s experiences and risk factors for IPV, including power imbalances in intimate-partner and carer relationships and barriers to accessing specialist support services [16, 45].

This study contributes to the social model understanding of disability by highlighting how gender, as a social and contextual factor, may interact with disability to shape experiences and risks of IPV, as suggested by the observed gendered patterns in our data.

The association between disability and IPV victimisation among women demonstrates the importance of gender-responsive, coordinated and complementary responses to IPV between specialist support services for victim-survivors, disability support services, and the health and social care system in the UK [46]. Detailed analysis of gendered associations between different types of disability and IPV supports the need to address challenges around less visible forms of IPV, intimate-partner carer reliance and power-imbalances, inadequate service funding [47] (particularly within the context of Austerity), and of specialist support provisions for women with complex needs [48].

In terms of the visibility of victimisation, this study found that disabled women (across all disability types) had higher odds of experiencing less visible forms of IPV—namely sexual and psychological IPV—while no significant association was observed for physical IPV. As well as demonstrating the importance of inclusive definitions of IPV and the distinction between different IPV types when exploring associations, this finding suggests a need for increased attention to identifying and addressing less visible forms of IPV, including psychological and sexual IPV, in the context of wider literature on accessible routes to disclosure and identification of victimisation among disabled women, such as via informed risk identification approaches through health and social care contact points [49].

Isolation and (in)visibility have been identified as key risk factors for IPV among disabled people, particularly in the context of power imbalances and dependence within intimate-partner carer relationships [14, 16]. Reductions in public funding for health and specialised services during Austerity have contributed to an increased reliance on familial and partner relationships to provide care for disabled people [50]. Whilst family and intimate partners provide invaluable care and support to disabled people, dependence on these relationships not only produces health inequalities based on financial and interpersonal-support disparities but also exacerbates power imbalances and isolates victims within abusive relationships due to a lack of alternative or alleviating care options [14, 16].

In demonstrating the heightened risk of IPV for disabled women, particularly in less visible forms, this study highlights the importance of accessible care and support outside of personal relationships, support and awareness-raising for carers regarding healthy relationships, and disclosure routes through independent and confidential contact with health and social care practitioners through both outreach efforts and within routine contact.

While our findings indicate that disability is not a significant risk factor for IPV among men, this does not diminish the importance of addressing the needs of male victims. The absence of a heightened risk associated with disability suggests that risk may be influenced by additional factors, such as socioeconomic conditions, mental health challenges, and societal stigma surrounding disclosure and help-seeking behaviours [51].

Inadequate funding of public health and care services is particularly concerning in light of our finding that socio-economic disadvantage is associated with all types of disability and a heightened risk of economic IPV among women with restricted ADLs. Furthermore, women with restricted ADLs were found to experience socio-economic disadvantage more frequently than both non-disabled women and women with other types of disabilities. These findings underscore the critical need for adequately funded and equitably distributed resources for health and social care services. This includes targeted support for disadvantaged groups with complex needs. Additionally, the intersection of restricted ADLs and socio-economic disadvantage emerges as a key indicator of increased vulnerability to economic IPV among women.

In responding to the further increased risk of IPV victimisation for women with *multiple* disability types found in this study, targeted funding may benefit victim and disability support services’ ability to cater to multiple, complex needs which account for gender and disability-based inequalities, as well as the specific needs of those with multiple disabilities, who often face additional barriers in accessing support which addresses diverse and interacting disabilities and limitations [48].

In summary, these findings highlight the need for a multi-agency response from gender and disability-responsive specialist victim-survivor services, and IPV risk-informed public health and social care services, underpinned by adequate funding, professional training, information sharing, clear referral pathways, quality assurance mechanisms and coordinated interventions.

This study demonstrates the significance of gender-disability intersections for IPV, providing compelling evidence to support the vital role of specialist interventions. Using nationally representative data from a high-quality population survey, it accounts for multiple types and dimensions of disability, employs stringent sampling and data collection methodologies, and enables analysis of associations between different forms of disability and IPV by gender.

### Limitations

To avoid conflating the effects of pre-existing psychological disabilities on IPV victimisation with the effects of IPV victimisation in causing or exacerbating psychological disabilities, psychological disabilities were excluded. To disentangle this relationship, longitudinal methods and temporal questionnaire designs are needed to better understand their causal mechanisms.

A limitation of our study is the potential impact of our disability definition on prevalence estimates. Our approach included individuals with restricted ADLs within the disability category, which may have contributed to the higher overall disability prevalence observed in our sample compared to some prior studies [27, 52, 53]. This broader definition means that individuals without specific impairments but with ADL restrictions were included, a population that may have been excluded from the disability group in other studies. However, our research, supported by others [54], highlights the importance of including this group in analyses, particularly considering their high reported rates of IPV. Future research should consider these definitional nuances to enhance consistency in understanding the intersection of disability types and IPV.

Further, by sampling households, our sample was potentially skewed towards relatively healthy individuals, excluding respondents from rest homes, hospitals, and refuges. This exclusion may have resulted in underrepresenting individuals who are more likely to be affected by disability and IPV. Thus, the prevalence of both disability and IPV and the strength of their associations may have been underestimated. In addition, IPV estimates reflect experiences within the past 12 months; while this approach improves temporal specificity, it may miss those whose most recent IPV exposure occurred outside this window.

Additionally, the relatively low response rate of the APMS could introduce bias, as non-respondents may systematically differ from those who participated. To address this, the weighting strategy accounted for multiple factors, including known area-level and property characteristics, as well as age and gender within regions. However, the risk of bias from non-response patterns remains a possibility and may affect the generalisability of the findings.

Another limitation of our study is the absence of specific measures of coercive control within the APMS dataset, which restricts our ability to fully capture the nuanced dynamics of coercive control and its association with different types of disability. This is important as previous research has demonstrated that coercive control is a distinct and pervasive form of IPV that often exerts a profound impact on victims, particularly those with disabilities [26, 55]. Moreover, use of a single-item measure of economic abuse potentially captured sporadic occurrences, rather than systematic patterns. Our definition of sensory disability was also limited to vision and hearing impairments and did not include less commonly measured senses such as touch, taste, or smell. “The ‘multiple disability types’ category may include respondents counted in more than one disability type due to differences in measurement thresholds across physical, sensory, learning, and ADL impairments. This overlap introduces some heterogeneity and potential double counting across disability measures.

The smaller sample size for disabled men may have contributed to the statistically non-significant findings and wide confidence intervals for sexual IPV, however, the consistent trends observed in the data suggest that disability may be a less relevant risk factor for men’s experience of IPV.

Finally, this research does not address the intersection of disability and other characteristics (than gender), including age, ethnicity, religion, and sexual orientation. Particularly, the nature and experience of disability may vary across the lifespan, especially as it becomes more ‘normalised’ in older age. Although we adjusted for age in all models, we did not stratify by age or include age–disability interaction terms, which may have limited our ability to detect age-specific effects. We were also unable to examine IPV experiences by sexual orientation due to small numbers of participants with both 12-month IPV exposure and specific disability types. This is an important limitation, as IPV prevalence may be higher in same-sex relationships [56], and future research should explore how disability intersects with sexual orientation and relationship type. Multiple identities may compound risk and vulnerability, leading to unique challenges in accessing support services, help-seeking, and escaping abusive situations. Research and interventions that consider intersections are essential for addressing survivor needs and promoting inclusive and accessible support systems.

## Conclusion

This study contributes new evidence of the significance of gender in the relationship between disabilities and IPV victimisation, disaggregated by disability and IPV types and controlling for sociodemographic and socioeconomic factors. It demonstrates the importance of nuanced understandings and highly specialised interventions for IPV that account for the interaction between gender and disability-based experiences and inequalities. The findings should be used to inform policies, service provision and resource allocation aimed at reducing IPV to better protect and support disabled women from violence.

## Supplementary Information

Supplementary Material 1

## Data Availability

No datasets were generated or analysed during the current study.
