# Supplementary material for: Disability and intimate partner violence in England: gender stratified analyses of a probability sample survey
Source: BMC Public Health. 2026 Jul 9;26:2921. doi: 10.1186/s12889-026-28489-5 (PMC13651323; doi:10.1186/s12889-026-28489-5)
Supplement: Supplementary file 1 [file 12889_2026_28489_MOESM1_ESM.docx]

**Appendix eTable 1. Questions’ wording, response options, and definition of variables**

| **Restricted Activities of Daily Living** | Restricted activities of Daily Living were measured using Activities of Daily Living (ADL) questionnaire. A number of different ADL were asked about including **personal care** such as dressing, getting out and about or using transport, medical care such as taking medicines or pills, having injections or changes of dressing, **household activities** like preparing meals, shopping, laundry, and household, **practical activities** such as gardening, decorating, or doing household repairs, **dealing with paperwork** such as writing letters, sending cards or filing forms, managing money, such as budgeting for food or paying bills. These are the activities that people need to perform on a daily basis to live independently. Respondents were asked whether they experience any difficulty with any of the ADLs. Response options included: “No, no difficulty at all”, “Yes, some difficulty”, and “Yes, a lot of difficulty”. A binary variable was created for experiencing “ a lot of difficulty” in performing any ADLs. |
| --- | --- |
| **Physical disability** | Participants were asked whether they had a health problem or a condition that has lasted ≥12 months and that reduced their ability to carry out day-to-day activities (in total 22 physical health conditions were asked about such as asthma, cancer, diabetes, epilepsy, and cardiovascular disease). Individuals were classified as having a physical disability if they said yes to at least one chronic physical health condition and that health condition reduced their ability to carry out day-to-day activities. |
| **Sensory disability** | Questions were asked of all participants about sight loss (ability to read newsprint at arms’ length or clearly seeing the face of someone across a room, with glasses if used) and about hearing loss (including whether or not hearing aids are used). Where sensory impairments were reported, level of impact on daily life was asked. A binary variable was created and those who reported that sight or hearing loss has limited (to some degree) the amount or kind of activities that they can do were identified as having sensory disability. |
| **Learning disability** | All respondents were asked whether they ‘have a difficulty learning or an intellectual disability’ (yes or no) and whether this impairment has limited the amount or kind of activities that they can do. Individuals were classified as having a learning disability if they said yes to learning impairment that has limited their daily activities. |
| **Any disability** | A binary variable was created for experiencing any disability (at least one versus none) |
| **Multiple disability types** | Respondents reporting two or more of the measured disability categories (physical, sensory, learning, or restricted ADLs). Note that individuals may be counted in more than one category, so this group includes overlapping impairments. |
| **Physical IPV** | Physical IPV was identified if either of the following were endorsed: “pushed you, held or pinned you down, or slapped you” or “kicked you, bit you, or hit you with a fist or something else, or threw something at you that hurt you” and if this happened in the past 12 months. |
| **Sexual IPV** | Sexual IPV was recorded if the participant responded yes to: “since the age of 16, has anyone touched you, or got you to touch them, in a sexual way without your consent?” or “since the age of 16, has anyone had sexual intercourse with you without your consent?”, or both, and if the perpetrator was a current or former partner and if this happened in the past 12 months. Variables were derived for having experienced any sexual violence from a partner (combining rape and non-consensual sexual contact). |
| **Economic IPV** | Economic IPV was recorded if the participant reported that a partner had “prevented you from having your fair share of the household money” and if this happened in the past 12 months. |
| **Psychological IPV** | Psychological IPV was identified where either of the following were endorsed: “repeatedly belittled you to the extent that you felt worthless” or “sent you more than one unwanted letter, email, text message, or card that was either obscene or threatening and which caused you fear, alarm, or distress” and if this happened in the past 12 months. |
| **Any IPV** | A binary variable was created for experiencing any 12-month IPV versus none. |
